# Crime and Violence among MDMA Users in the United States

**DOI:** 10.3934/publichealth.2015.1.64

**Published:** 2015-03-18

**Authors:** Michael G. Vaughn, Christopher P. Salas-Wright, Matt DeLisi, Brian E. Perron, David Cordova

**Affiliations:** 1 School of Social Work, College for Public Health and Social Justice, Saint Louis University, Saint Louis, MO 63103, United States; 2 School of Social Work, The University of Texas at Austin, Austin, TX 78712, United States; 3 Criminology and Criminal Justice Studies, Department of Sociology, Iowa State University, Ames, IA 50013, United States; 4 School of Social Work, University of Michigan, Ann Arbor, MI 48109, United States

**Keywords:** ecstasy, MDMA, crime, violence, drug use, gender

## Abstract

The question of whether MDMA use is associated with increased crime and violence has not been adequately explored especially in nationally representative samples. This study used data from the National Epidemiologic Survey on Alcohol and Related Conditions (NESARC) to assess the association between MDMA use and violent and non-violent antisocial behavior while controlling for sociodemographic variables, lifetime psychiatric, alcohol and drug use disorders, and family history of antisocial behavior. MDMA users, both male and female, were involved in a number of crimes in acts of violence including drunk driving, shoplifting, theft, intimate partner violence, and fighting. Notably, female MDMA users were more antisocial than male non-MDMA users. Although adjusting the results for numerous confounds attenuated the relationships, MDMA users were still at significantly greater odds of engaging in violent and nonviolent crime than non-MDMA users. Although MDMA has been considered a facilitator of empathy and closeness, the current study suggests a dark side as MDMA is associated with a broad array of crimes and transgressions. Additional tests of the MDMA-crime link are needed to properly inform policy.

## 1. Introduction

MDMA (3,4 methylene-dioxymetamphetamine), also known as Ecstasy is a phenethylamine that is similar to both amphetamine and methamphetamine [1],[2]. MDMA possesses potent stimulant qualities but is different from amphetamines and methamphetamine in that MDMA has a particular affinity for the serotonin transporter [2]. MDMA was first synthesized nearly one hundred years ago (1912) and due to its purported ability to elicit empathy gained some initial notoriety as an adjunct used during couples therapy in the 1970's. MDMA became popular as a street drug in the 1980's and was made illegal in 1985 [3]. MDMA is usually taken in tablet form with a standard dose of 0.75–4.0 mg per kilogram of body weight [4]. MDMA users report rapid onset, euphoria, added energy, and enhanced closeness to others [5],[6]. Despite these pleasurable effects, users can also report anxiety and irritability, impulsiveness, paranoia, muscle cramps, potentially fatal hyperthermia, and mood changes that heighten aggression [1],[7]–[9].

In 1990's and early 2000's there was an alarming rise in the availability and use of synthetic “club drugs” most notable of which is Ecstasy (MDMA). The use of ecstasy at large youth parties known as “raves” has garnered an abundance of electronic and print media attention. Several recent studies of MDMA-assisted psychotherapy for post-traumatic stress disorder have emerged showing possible promising results [10].

At various junctures in time, the cultural context of MDMA has changed from being considered a love, hug, and general party drug. More recent research suggests, however, that MDMA users may also be engaged in relatively high levels of violent and non-violent crime. Reid and colleagues [11] found a connection between MDMA use and aggression among 260 young adult MDMA users. Specifically, young adult MDMA users who were most aggressive were those low on a measure of self-control suggesting that impulsivity is the behavioral mechanism by which MDMA is linked to aggression.

Insufficient research exists relative to the nature of MDMA use and crime and violence. In a Scottish study of 209 participants recruited from dance clubs, Hammersley et al. [12], found MDMA users were involved in a wide range of illegal activities but also commonly used other illicit substances. Yacoubian et al. [13] collected self-report drug use data and urine specimens from 209 youthful offenders and found that 16% reported using MDMA within the past year, which is significantly higher than non-offending youth. In a prospective longitudinal investigation of four years Lieb et al. [14], concluded that mental health disorders are associated with multiple substances including MDMA. Confounding of prior and current mental health problems and substance abuse underscores the difficulty in identifying a relationship between MDMA use and crime given that the vast majority of MDMA users evince a polydrug use career. In addition, generalizability is an issue as there have been no studies of MDMA use and crime in population- based samples.

The purpose of the present study is to surmount prior limitations in examining the MDMA-crime link. We do so by employing data sourced from the National Epidemiologic Survey of Alcohol and Related Conditions (NESARC). NESARC is a nationally representative sample that is ideally suited to the present study due to its generalizability and extensive assessment of drug use, mental health disorders, and antisocial behavior. We hypothesize that MDMA use will be associated with both violent and non-violent crime even after controlling for notable confounds such as alcohol and other illicit drug use, mental health disorders, and sociodemographic characteristics.

## 2. Methods

Study findings are based on data from Waves I (2001–2002) and II (2004–2005) of the National Epidemiologic Survey of Alcohol and Related Conditions (NESARC). The NESARC is a nationally representative sample of non-institutionalized U.S. residents aged 18 years and older. The NESARC utilized a multistage cluster sampling design, oversampling young adults, Hispanics, and African-Americans in the interest of obtaining reliable statistical estimation in these subpopulations, and to ensure appropriate representation of racial/ethnic subgroups. Data were collected through face-to-face structured psychiatric interviews conducted by U.S. Census workers trained by the National Institute on Alcohol Abuse and Alcoholism and U.S. Census Bureau. Data were weighted at the individual and household levels to adjust for oversampling and non-response on demographic variables (i.e., age, race/ethnicity, sex, region, and place of residence). Data were also adjusted to be representative (based on region, age, race, and ethnicity) of the U.S. adult population as assessed during the 2000 Census. The U.S. Census Bureau and the U.S. Office of Management and Budget approved the research protocol and informed consent procedures. The response rate for Wave I data was 81% and for Wave II was 87% with a cumulative response rate of 70% for both waves. Based on the distribution of MDMA users in the general population, the current study restricted analyses to adults between the ages of 18 and 49 (*n* = 19,073). A more detailed description of the NESARC design and procedures is available elsewhere [15].

### 2.1. Measures

MDMA Users. Respondents were asked, “Have you ever used ecstasy or MDMA?” Data from Waves I and II were combined to measure respondent self-report of lifetime Ecstasy/MDMA use (0 = no, 1 = yes).

#### 2.1.1. Crime and Violence

Twelve dichotomous (0 = no, 1 = yes) measures from the antisocial personality disorder module of the Alcohol Use Disorder and Associated Disabilities Interview Schedule––DSM-IV version (AUDADIS-IV) were used to examine criminal and violent behavior. Data from Waves I and II were combined to measure respondent self-report of having exhibited any of the behaviors in their lifetime. In addition to the twelve single-item measures, we also created two additional dichotomous measures of involvement in any of the criminal and violent behaviors examined in the study. Specifically, individuals who responded affirmatively to one or more of the criminal behavior variables were coded as 1 while those who did not respond affirmatively to any of the criminal behavior variables were coded as 0. An identical coding procedure was implemented with respect to any lifetime involvement in violent behavior (0 = no lifetime involvement in any violent behavior, 1 = lifetime involvement in one or more violent behaviors). Only variables measuring nonviolent criminal and violent behaviors with prevalence greater than 3% were included in statistical analyses.

#### 2.1.2. Sociodemographic and Behavioral Controls

The following demographic variables were included as controls: age, gender, race/ethnicity, household income, education level, marital status, region of the United States, and urbanicity. To better isolate the link between MDMA use and crimogenic variables we also controlled for parental history of antisocial behavior, parental substance use problems, lifetime use of other licit or illicit substances (i.e., alcohol, cannabis, cocaine/crack, amphetamines, inhalants, tranquilizers, and heroin) and lifetime diagnoses of clinical and personality disorders.

### 2.2. Data Analysis

A series of logistic regression analyses were conducted that compared the criminal and violent behavior of MDMA users with non-users while controlling for aforementioned variables. Stratified logistic regression was carried out to examine the links between MDMA use and crime/violence across gender. Weighted prevalence estimates and associated 95% confidence intervals were computed using Stata 13.1 SE software [16]. This system implements a Taylor series linearization to adjust estimates for complex survey sampling design effects including clustered data. Estimates for all analyses were obtained using Wave 2 weights. Additional information regarding the weighting procedures utilized in the analyses of NESARC data is available elsewhere [17]. Adjusted odds ratios (AORs) were considered to be statistically significant if the associated confidence intervals did not cross the 1.0 threshold.

## 3. Results

Table 1 displays the sociodemographic characteristics of individuals between the ages of 18 and 49 reporting having ever used MDMA. Compared to nonusers, individuals reporting having used MDMA were significantly more likely to be male (AOR = 1.69, 95% CI = 1.57–1.83), to reside in a household earning less than $20,000 per year (AOR = 1.44, 95% CI = 1.24–1.67), to have completed some college (AOR = 1.20, 95% CI = 1.11–1.29), and to be either separated/divorced (AOR = 1.68, 95% CI = 1.40–2.03) or never married (AOR = 1.88, 95% CI = 1.73–2.05). MDMA users were significantly less likely to be between the ages of 18 and 34 (AOR = 0.23, 95% CI = 0.20–0.26), to be either African-American (AOR = 0.14, 95% CI = 0.12–0.17) or Hispanic (AOR = 0.64, 95% CI = 0.57–0.72), to have graduated from high school only (AOR = 0.86, 95% CI = 0.78–0.94) and to reside in a region other than the Western United States. No significant differences were observed in terms of urbanicity.

**Figure 1.**
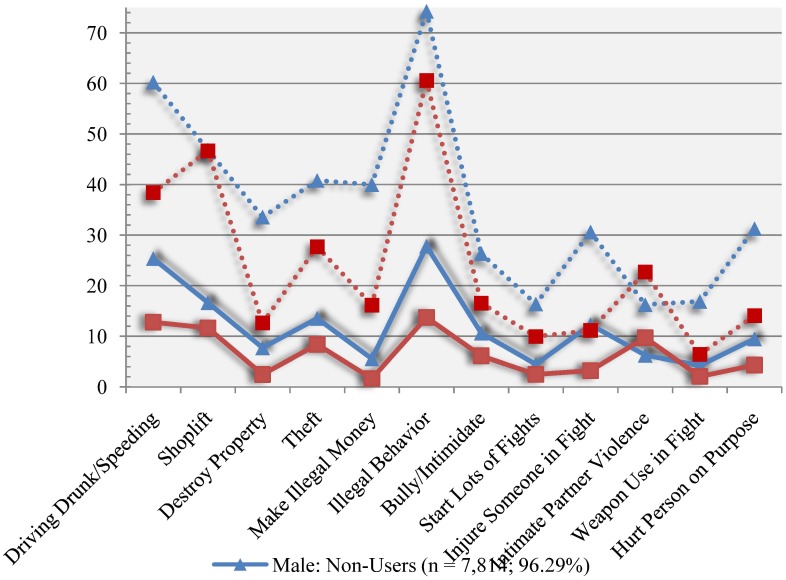
Prevalence of crime and violence among MDMA users in the United States.

Figure 1 displays the lifetime prevalence of criminal and violent behavior among male and female MDMA users and nonusers. Across gender, the prevalence of criminal and violent behavior was greater among MDMA users compared to non-MDMA users. Moreover, with the exception of injuring someone in a fight, the prevalence of crime and violence among female MDMA users was greater than that of male nonusers. With the exception of intimate partner violence, the prevalence of all of criminal and violent behaviors was greater among male MDMA users compared to female MDMA users.

Table 2 compares the prevalence of violent and criminal behavior among MDMA users in contrast with nonusers. Controlling for sociodemographic factors, parental antisocial and substance use characteristics, lifetime substance use, and psychiatric morbidity, MDMA users were significantly more likely to report involvement in all criminal and violent behaviors examined in this study. Supplementary stratified logistic regression analyses yielded additional information with respect to the behaviors of MDMA users across gender. With respect to crime, robust effects were observed for both women (AOR = 1.94, 95% CI = 1.64–2.31) and men (AOR = 1.77, 95% CI = 1.47–2.14); however, while the odds ratio was slightly larger for women, no significant differences in effects were observed. Significant gender differences were observed in terms of the relationship between MDMA use and violence. Namely, while male MDMA users were significantly more likely to enact violence (AOR = 1.73, 95% CI = 1.51–2.00), female MDMA users were found to be significantly less likely to enact violence compared to female nonusers when controlling for sociodemographic factors, parental antisocial and substance use characteristics, lifetime substance use, and psychiatric morbidity (AOR = 0.77, 95% CI = 0.63–0.94).

**Table 1.** Sociodemographic characteristics of MDMA users in the United States.

Sociodemographic Factors	Ever used ecstasy or MDMA?				Unadjusted		Adjusted	
	No (*n* = 18,548; 96.80%)		Yes (*n* = 519; 3.20%)					
	%	95% CI	%	95% CI	OR	(95% CI)	OR	(95% CI)
*Age*								
18–34 years	43.80	(43.3–44.3)	80.51	(78.7–82.2)	**0.19**	**(0.17–0.21)**	**0.23**	**(0.20–0.26)**
35–49 years	56.20	(55.7–56.7)	19.49	(17.8–21.3)	1.00		1.00	
*Gender*								
Female	50.99	(50.5–51.4)	37.00	(35.5–38.5)	1.00		1.00	
Male	49.01	(48.6–49.4)	63.00	(61.4–64.5)	**1.77**	**(1.66–1.89)**	**1.69**	**(1.57–1.83)**
*Race/Ethnicity*								
Non-Hispanic	65.30	(64.6–65.9)	75.01	(73.0–76.9)	1.00		1.00	
White								
African	12.53	(12.0–13.0)	2.70	(2.3–3.1)	**0.19**	**(0.16–0.22)**	**0.14**	**(0.12–0.17)**
American								
Hispanic	**6.94**	(6.7–7.2)	9.15	(7.8–10.6)	**0.75**	**(0.68–0.83)**	**0.64**	**(0.57–0.72)**
Other	15.23	(14.9–15.6)	13.14	(12.3–14.0)	1.15	(0.95–1.39)	1.04	(0.86–1.27)
*Household Income*								
< $20,000	16.12	(15.7–16.5)	25.53	(23.5–27.6)	**1.99**	**(1.75–2.27)**	**1.44**	**(1.24–1.67)**
$20,000–$34,999	17.46	(17.1–17.8)	18.64	(16.8–20.6)	**1.34**	**(1.16–1.56)**	1.09	(0.94–1.7)
$35,000–$69,999	34.02	(33.6–34.4)	30.10	(28.6–31.6)	**1.11**	**(1.01–1.23)**	0.96	(0.86–1.07)
> $70,000	32.40	(32.0–32.8)	25.73	(23.9–27.6)	1.00		1.00	
*Education Level*								
Less than H.S.	11.30	(11.0–11.6)	10.18	(8.7–11.8)	0.99	(0.84–1.17)	0.92	(0.76–1.12)
H.S. Graduate	25.33	(24.8–25.9)	20.35	(18.8–22.0)	**0.88**	**(0.79–0.99)**	**0.86**	**(0.78–0.94)**
Some College	24.07	(23.7–24.4)	33.79	(30.2–35.4)	**1.55**	**(1.43–1.67)**	**1.20**	**(1.11–1.29)**
Completed AA								
BA, or Technical	39.30	(38.8–39.8)	35.68	(34.0–37.4)	1.00		1.00	
Degree								
*Marital Status*								
Married/	62.66	(62.2–53.1)	39.42	(37.9–41.0)	1.00		1.00	
Cohabitating	10.75	(10.5–11.1)	9.20	(7.9–10.7)	**1.36**	**(1.14–1.63)**	**1.68**	**(1.40–2.03)**
Separated/Divorced								
Widowed	0.58	(0.52–0.65)	0.16	(0.15–0.17)	**0.43**	**(0.38–0.50)**	0.73	(0.48–1.10)
Never Married	26.00	(25.5–26.5)	51.22	(49.6–52.9)	**3.13**	**(2.91–3.36)**	**1.88**	**(1.73–2.05)**
*Region of U.S.A*.								
West	17.19	(16.7–17.6)	15.41	(14.3–16.6)	1.00		1.00	
Northeast	18.50	(18.1–18.9)	18.32	(16.8–20.0)	**0.70**	**(0.61–0.79)**	**0.72**	**(0.63–0.82)**
Midwest	39.20	(38.7–39.7)	33.94	(31.9–36.0)	**0.77**	**(0.67–0.88)**	**0.78**	**(0.67–0.90)**
South	25.11	(24.7–25.5)	32.33	(30.4–34.3)	**0.67**	**(0.60–0.75)**	**0.62**	**(0.55–0.70)**
*Urbanicity*								
Rural	67.55	(66.9–68.2)	68.76	(67.5–70.0)	1.00		1.00	
Urban	32.45	(31.8–33.1)	31.24	(30.0–32.5)	**0.95**	**(0.89–1.00)**	0.97	(0.90–1.05)

**Note:** Adjusted odds ratios adjusted for age, race/ethnicity, household income, education level, region of the United States, and urbanicity. Odds ratios and confidence intervals in bold are statistically significant.

**Table 2.** Crime and Violence among MDMA users in the United States.

	Ever used ecstasy or MDMA?				Unadjusted		Adjusted	
	No (*n* = 18,548; 96.80%)		Yes (*n* = 519; 3.20%)					
	%	95% CI	%	95% CI	OR	(95% CI)	AOR	(95% CI)
**Crime**								
Do things that could have easily hurt you or someone else – like speeding or driving after having too much to drink?								
No	81.06	(80.7–81.4)	47.81	(45.9–49.7)	1.00		1.00	
Yes	18.94	(18.6–19.3)	52.19	(50.3–54.1)	**4.67**	**(4.29–5.09)**	**1.40**	**(1.25–1.56)**
Shoplift?								
No	85.90	(85.6–86.2)	53.22	(51.4–35.0)	1.00		1.00	
Yes	14.10	(13.8–14.4)	46.78	(45.0–48.6)	**5.35**	**(4.96–5.78)**	**1.27**	**(1.14–1.42)**
Steal anything from someone or someplace when no one was around?								
No	89.09	(88.8–89.4)	64.04	(62.3–65.7)	1.00		1.00	
Yes	10.91	(10.6–11.2)	35.96	(34.3–37.7)	**4.58**	**(4.23–4.97)**	**1.44**	**(1.28–1.63)**
Destroy, break, or vandalize someone else's property?								
No	94.96	(94.7–95.1)	74.19	(72.1–76.2)	1.00		1.00	
Yes	5.04	(4.8–5.2)	25.81	(23.8–27.9)	**6.55**	**(5.85–7.33)**	**1.53**	**(1.28–1.81)**
Made money illegally like selling stolen property or selling drugs?								
No	96.44	(96.2–96.6)	68.85	(66.9–70.7)	1.00		1.00	
Yes	3.56	(3.4–3.7)	31.15	(29.3–33.0)	**12.2**	**(11.2–13.4)**	**1.64**	**(1.41–1.91)**
Do anything that you could have been arrested for?								
No	79.38	(78.9–79.8)	30.78	(29.2–32.5)	1.00		1.00	
Yes	20.62	(20.2–21.0)	69.22	(67.5–70.8)	**8.66**	**(8.03–9.34)**	**1.58**	**(1.42–1.76)**
								
**Violence**								
Bullied or pushed people around or tried to make them afraid of you?								
No	91.65	(91.4–91.9)	77.28	(75.1–79.3)	1.00		1.00	
Yes	8.35	(8.1–8.6)	22.72	(20.7–24.9)	**3.23**	**(2.84–3.67)**	**1.21**	**(1.02–1.45)**
Get into a lot of fights that you started?								
No	96.54	(96.3–96.7)	85.99	(84.4–87.5)	1.00		1.00	
Yes	3.46	(3.3–3.6)	14.01	(12.5–15.6)	**4.54**	**(3.95–5.22)**	**1.34**	**(1.08–1.66)**
Hit someone so hard that you injure them or they had to see a doctor?								
No	92.35	(92.0–92.7)	76.64	(74.7–78.5)	1.00		1.00	
Yes	7.65	(7.3–8.0)	23.36	(21.5–25.3)	**3.68**	**(3.28–4.13)**	**1.24**	**(1.03–1.49)**
Get into a fight that came to swapping blows with romantic partner?								
No	91.97	(91.7–92.2)	81.34	(80.1–82.5)	1.00		1.00	
Yes	8.03	(7.8–8.3)	18.66	(17.5–19.9)	**2.63**	**(2.41–2.86)**	**1.28**	**(1.13–1.45)**
Use a weapon like a stick, knife, or gun in a fight?								
No	96.88	(96.7–97.0)	86.98	(85.4–88.4)	1.00		1.00	
Yes	3.12	(3.0–3.3)	13.02	(11.6–14.6)	**4.64**	**(4.00–5.39)**	**1.98**	**(1.65–2.36)**
Physically hurt another person in any way on purpose?								
No	93.15	(92.9–93.4)	75.07	(73.0–77.0)	1.00		1.00	
Yes	6.85	(6.6–7.1)	24.93	(23.0–26.9)	**4.51**	**(4.01–5.08)**	**1.46**	**(1.23–1.73)**

**Note:** Adjusted odds ratios adjusted for age, gender, race/ethnicity, household income, education level, marital status, region of the United States, urbanicity, parental history of antisocial behavior and substance abuse history, lifetime substance use (alcohol, cannabis, cocaine/crack, amphetamines, inhalants, tranquilizers, and heroin) and lifetime diagnosis of any clinical or personality disorder.

## 4. Discussion

Our objective was to examine the association between MDMA and crime and violence and assess the robustness of the relation by controlling for numerous confounds. To our knowledge, this is the largest study ever conducted on MDMA and crime. We found that MDMA users, both male and female, were involved in a number of crimes in acts of violence including drunk driving, shoplifting, theft, intimate partner violence, and fighting. Notably, female MDMA users were more antisocial than male non-MDMA users. Although adjusting the results for numerous confounds attenuated the relationships, MDMA users were still at significantly greater odds of engaging in violence and nonviolent crime than non- MDMA users. These findings support prior research that indicated that MDMA is associated with aggression [11]. Given that violence has been established as a major health concern, it is important to point out illicit drug is linked to both violence and poor health. Although MDMA use is substantially less than that of alcohol and other substances found to be associated with violence, it nevertheless is a contributor to the drugs-violence public health nexus.

It is not entirely clear as to the mechanism(s) by which MDMA is associated with crime and violence. Reid and colleagues [11] found that MDMA users were more impulsive and therefore more likely to be reactively aggressive. Investigations on adults who use MDMA suggest that this drug generates persistent damage to serotonin-releasing neurons[1] and that MDMA is a powerful selective serotonin neurotoxin [18],[19]. Multiple studies have found psychiatric disorders such as anxiety and depression is relatively common among MDMA users [20]–[22]. Serotonin transporter dysfunction has been linked to violence in several studies [23]. It could also simply be the case that individuals with difficult temperaments are more likely to use MDMA and be anger and crime-prone [24].

Despite the many assets of the study, several limitations should be noted. One limitation is the data are cross-sectional. Although we control for a substantial number of confounds, we are unable to clarify the temporal ordering of associations in the data. Thus, the causal status of MDMA use and crime and violence is not established. Moreover, we do not know the long-term status that MDMA use has on crime and violence. This will require data from prospective longitudinal designs. An additional limitation is that the data did not include important contextual information (e.g., situations of use) which could be used in understanding the MDMA-crime connection. Future studies on MDMA should consider these data features.

## 5. Conclusion

Like many drugs of abuse, MDMA has had a multifaceted career. Whether thought of as a facilitator of empathy and closeness (i.e., love and hugs) or as a pathway to crime and violence (i.e., mugging), new research on the behavioral effects of MDMA are needed to clarify its proper role. The current study suggests that MDMA is associated with a broad array of crimes and transgressions at the population-level for both male and female users. Although additional tests of the MDMA-crime link are needed to properly inform policy, findings from this national study suggest that there are public health consequences to the proliferation and ingestion of MDMA.
